# The *Anabaena* sp. PCC 7120 Exoproteome: Taking a Peek outside the Box

**DOI:** 10.3390/life5010130

**Published:** 2015-01-08

**Authors:** Paulo Oliveira, Nuno M. Martins, Marina Santos, Narciso A. S. Couto, Phillip C. Wright, Paula Tamagnini

**Affiliations:** 1 Instituto de Investigação e Inovação em Saúde, Universidade do Porto, Porto 4150-180, Portugal; E-Mails: nderesen@ulb.ac.be (N.M.M.); marina.santos@ibmc.up.pt (M.S.); pmtamagn@ibmc.up.pt (P.T.); 2 IBMC-Instituto de Biologia Molecular e Celular, Universidade do Porto, Porto 4150-180, Portugal; 3 Laboratoire du Biologie du Noyau, Institut de Biologie & de Médecine Moléculaire, Université Libre de Bruxelles, Charleroi B-6041, Belgium; 4 ChELSI Institute, Chemical and Biological Engineering, University of Sheffield, Sheffield S1 3JD, UK; E-Mails: n.couto@sheffield.ac.uk (N.A.S.C.); p.c.wright@sheffield.ac.uk (P.C.W.); 5 Faculdade de Ciências, Departamento de Biologia, Universidade do Porto, Porto 4169-007, Portugal

**Keywords:** cyanobacteria, exoprotein, protein secretion, membrane vesicles

## Abstract

The interest in examining the subset of proteins present in the extracellular milieu, the exoproteome, has been growing due to novel insights highlighting their role on extracellular matrix organization and biofilm formation, but also on homeostasis and development. The cyanobacterial exoproteome is poorly studied, and the role of cyanobacterial exoproteins on cell wall biogenesis, morphology and even physiology is largely unknown. Here, we present a comprehensive examination of the *Anabaena* sp. PCC 7120 exoproteome under various growth conditions. Altogether, 139 proteins belonging to 16 different functional categories have been identified. A large fraction (48%) of the identified proteins is classified as “hypothetical”, falls into the “other categories” set or presents no similarity to other proteins. The evidence presented here shows that *Anabaena* sp. PCC 7120 is capable of outer membrane vesicle formation and that these vesicles are likely to contribute to the exoproteome profile. Furthermore, the activity of selected exoproteins associated with oxidative stress has been assessed, suggesting their involvement in redox homeostasis mechanisms in the extracellular space. Finally, we discuss our results in light of other cyanobacterial exoproteome studies and focus on the potential of exploring cyanobacteria as cell factories to produce and secrete selected proteins.

## 1. Introduction

Cyanobacteria are prokaryotes that distinctively perform oxygenic photosynthesis. However, the phylum is highly heterogeneous, including strains that are morphologically diverse and metabolically plastic, and some even present the capacity of cellular differentiation. Given their minimal nutritional requirements, cyanobacteria can be found in a wide range of habitats and ecological niches, mainly free-living in aquatic environments, but they can also be found in extreme environments, as well as in symbiotic associations. Their cosmopolitan ecological distribution and their long evolutionary history are strong arguments for placing cyanobacteria among the most successful group of microorganisms.

The total collection of proteins in a cell, tissue or organism, under specific conditions of time, space and environment represents the proteome [1]. Several aspects of the cyanobacterial proteome have been carefully examined over the years. The initial studies aiming at identifying the maximum amount of proteins present in cyanobacteria [2,3] quickly led to more sophisticated analyses of the proteome dynamics in response to changes in environmental cues (e.g., [4,5,6,7]), as well as to the result of deleting or over-expressing a particular gene of interest (e.g., [8,9,10]). Several cyanobacterial strains have been the focus of proteomic studies, including *Synechocystis*, *Anabaena*, *Nostoc*, *Cyanothece*, *Gloeothece* and *Euhalothece* [5,6,11,12,13,14,15,16,17]. In addition, the protein composition of various subcellular compartments plus their dynamics in response to environmental and/or genetic changes has also been described, including in the outer-membrane [18,19], periplasm [20], cytoplasmic membrane [21,22], cytosol [23] and thylakoid membrane [9,24,25]. Additionally, various reports corroborate that cyanobacteria have the capacity of translocating proteins from the cytoplasm to the extracellular space (e.g., [26,27,28,29,30,31]). Once translocated across the outer membrane, a protein can remain anchored to the membrane, associate (non)-covalently with outer membrane components, assemble into macromolecular structures on the cell surface or be released into the surrounding environment [32].

The subset of proteins present in the extracellular milieu, the exoproteome, has been poorly studied in cyanobacteria: not only is the number of cyanobacterial strains examined restricted (*Synechocystis*, *Anabaena* and *Nostoc*), but also the growth conditions tested are limited [26,27,30]. Some difficulties arise when studying the exoproteome: many of the exoproteins of interest are transported to the extracellular milieu as the result of active protein secretion via a specific secretion system (secretome). Nevertheless, it may also be possible that cytosolic proteins or even membrane proteins end up accumulating in the extracellular space as the result of cell lysis, being relatively stable to proteolytic cleavage and, thus, contributing to the exoproteome. Thus, as pointed out by Desvaux *et al.* [32], an exoprotein is not necessarily translocated. Therefore, the major challenge is comprised of distinguishing between exoproteins that are secreted from those that accumulate in the extracellular milieu, but that are not actively transported. One strategy to address this challenge is to eliminate the cell’s protein secretion systems and evaluate which exoproteins are not transported. Such an analysis has already been adopted for *Anabaena* sp. PCC 7120 [26]. In that work, Hahn *et al.* [26] report on the secretome analysis and the involvement of the TolC-like protein, HgdD, a type I secretion system component, on protein secretion.

With the present work, we aim to profile the cyanobacterial exoproteome in the direction of unveiling novel cyanobacterial extracellular processes implicated in cell wall biogenesis, morphology and physiology. Therefore, here, we report on the isolation and identification of the exoproteome of *Anabaena* sp. PCC 7120 grown in medium with different nitrogen sources. Remarkably, a significant number of proteins were identified, belonging to various functional categories. In the present work, in addition to validating the mass spectrometry protein identifications by assessing some of the respective enzymes’ activities, we also analyse the potential of using cyanobacterial protein secretion for biotechnological applications.

## 2. Experimental Section

Organism and growth conditions: The filamentous, heterocyst-forming cyanobacterium *Anabaena* sp. PCC 7120 (also known as *Nostoc* sp. PCC 7120) was grown in liquid BG11 [33], BG11_0_ (BG11 medium without nitrate) or BG11_0_ supplemented with 5 mM NH_4_Cl and 10 mM HEPES-NaOH, pH 7.5 (BG11_0_ + NH_4_^+^), under a continuous light regime of 30–40 µmol photons m^−2^ s^−1^, at 28 °C. For exoproteome sample collection and concentration, cultures of *Anabaena* sp. PCC 7120 were inoculated with pre-cultures that had been growing in 100-mL Erlenmeyer flasks containing approximately 50 mL of culture volume. To minimize filament shearing and mechanical cell lysis, these pre-cultures were maintained either in static conditions or in an orbital shaker under mild shaking conditions (60–80 rpm). Pre-cultures were used as the inoculum when a final chlorophyll *a* concentration of 5–10 µg mL^−1^ was reached (approximately the same concentration as cultures should reach for exoproteome recovery and concentration; see below). Then, an aliquot of 4 mL of pre-culture was diluted with fresh growth medium to a final culture volume of 400 mL (1:100 dilution) in 500-mL glass gas washing bottles. Cultures were grown with aeration with air (flux of approximately 1 L min^−1^). In the case of strain BSMPo1, the *Anabaena* sp. PCC 7120 strain over-expressing *hesF* under the control of the *nirA* promoter [34], cultivation was done in BG11 medium supplemented with 30 µg mL^−1^ of neomycin.

Exoproteome isolation and analysis: The exoproteome of *Anabaena* sp. PCC 7120 was isolated as described previously [34]. Briefly, between 100 and 200 mL of *Anabaena* sp. PCC 7120 cultures grown in BG11, BG11_0_ or BG11_0_ + NH_4_^+^ to a final chlorophyll *a* concentration of approximately 8–10 µg mL^−1^ were collected by centrifugation (4000 ×*g*). The supernatants were filtered through 0.2-µm pore size filters and further concentrated by centrifugation with Amicon Ultra-15 Centrifugal Filter units (Merck Millipore) with a nominal molecular weight limit of 3 kDa. Concentrated exoproteome samples were either used immediately for zymographic and enzyme activity analyses (see below) or saved at −20 °C until further analysis. Exoproteome samples were separated by electrophoresis on SDS (sodium dodecyl sulphate)-polyacrylamide gels: in brief, electrophoresis was performed on a vertical mini Protean system (Bio-Rad, Hercules, CA, USA) according to the method of Laemmli [35]. A 10% gel was prepared, and samples that were mixed with Laemmli sample buffer and heated at 95 °C for 5 min were loaded on the gel. Protein separation was carried out at 20 mA. After protein separation, proteins were visualized using colloidal Coomassie Brilliant Blue G (Sigma-Aldrich, Poole Dorset, UK).

In-gel digestion: In-gel digestion was performed according to Shevchenko *et al.* [36] with some minor modifications. Briefly, Coomassie Blue-stained bands, observed consistently across at least 3 biological replicates, were excised and cut into cubes (*ca*. 1 × 1 mm), which were de-stained by successive washes and incubations (20 min) with a 50:50 (v/v) solution of 100 mM ammonium bicarbonate and acetonitrile. After the reduction and alkylation steps, digestion was performed in the presence of 0.2 μg of trypsin (Promega, Fitchburg, WI, USA) for each gel piece. After digestion, peptides were extracted with 50 μL of 5% (v/v) acetonitrile, 0.1% (v/v) formic acid and then with 50 μL of 50% (v/v) acetonitrile 0.1% (v/v) formic acid solution. The combined extracts were concentrated in a vacuum centrifuge at room temperature and stored at −20 °C until mass spectrometric analysis.

High-performance liquid chromatography (HPLC) mass spectrometry (MS) analysis: Extracted peptides were reverse-phase separated on an Ultimate 3000 capillary HPLC system (Dionex, Surrey, UK) and MS analysed on an Amazon ion trap mass spectrometer (Bruker, Bremen, Germany) interfaced with and electrospray ion source. On the LC system, a 75 μm × 15 cm C18 analytical column (LC Packings) preceded by a C18 trap column (LC Packings) was used to separate peptides at 30 °C using a flow rate of 300 nL min^−1^. Aqueous buffer (Buffer A, 3% (v/v) HPLC acetonitrile and 97% (v/v) HPLC water incorporating 0.1% (v/v) formic acid) and organic buffer (Buffer B, 97% (v/v) HPLC acetonitrile and 3% (v/v) HPLC water incorporating 0.1% (v/v) formic acid) were used to perform reverse-phase separation using a 90-min binary gradient started with 0% Buffer B for 5 min, followed by a linear ramp from 5% to 40% (v/v) Buffer B for 70 min, then an isocratic wash with 90% (v/v) Buffer B for 7 min and column re-equilibration at 5% (v/v) Buffer B for 8 min. Between every two sample injections, the HPLC ran a 40-min isocratic wash with 100% Buffer A to act as a blank and purge the system. LC eluate was sprayed on the MS using a capillary voltage of 2700 V, an end plate offset of 500 V, a dry gas temperature of 180 °C and a dry gas of 6 L min^−1^. The MS was set to detect positive ions using the standard enhanced mode with an m/z range of 200–2000. Ions were accumulated in the trap until the ion charge count (ICC) reached 20,000 with a maximum accumulation time of 200 ms. Peptide sequence information was inferred by collision-induced dissociation (CID) tandem mass spectrometry (MS/MS) experiments in a data-dependent acquisition fashion by selecting auto MS(n), where the top four most intense peaks were chosen for dissociation with a total ion count (TIC) absolute threshold of 25,000 and a relative threshold of 5% of the base peak.

MS/MS database search: After acquisition, raw data were converted into a mascot generic file (mgf) using DataAnalysis™ software, Version 4.2 (Bruker, Bremen, Germany). These peak lists containing the smoothed and centroided m/z and signal intensities were used for peptide identification using the EasyProt search algorithm [37] (developed between the Biomedical Proteomics Research Group of the Human Protein Science department at the University of Geneva, the Swiss Center for Applied Human Toxicology and the Swiss Institute of Bioinformatics) and the *Anabaena* sp. PCC 7120 protein database available at and downloaded from UniProt (http://www.uniprot.org/; accessed in July 2013). On EasyProt, trypsin was set as the enzyme, allowing for two missed cleavages; carbamidomethyl cysteine was considered as a fixed modification and oxidation of methionine as variable modification. The peptide tolerance and MS/MS tolerance were set to 0.6 Da. Data files containing CID were searched using the ESI-trap. Data quality was filtered, allowing only peptide identification with a Z-score >5 and a minimum length of 6 amino acids. To determine which proteins were present in the sample, a false discovery rate of no more than 1% was chosen, calculated using a reverse target-decoy [38] automatically generated by EasyProt. A further stringency of a minimum of two peptides identified per protein was applied. Subcellular localization prediction of proteins identified in the different exoproteomes was carried out using the online tool, PSORTb version 3.0 [39].

Superoxide dismutase (SOD) zymography and catalase activity analyses: Total protein extractions from *Anabaena* sp. PCC 7120 were performed using an extraction buffer containing either 50 mM Tris-HCl pH 7.5, 1 mM EDTA, 2 mM DTT, 10% glycerol and supplemented with a protease inhibitor cocktail (cOmplete Mini, EDTA-free, Roche, Basel, Switzerland) [40] for SOD measurements or 50 mM potassium phosphate pH 7.0 for catalase activity determination. In both cases, cells were lysed by sonication (Branson sonifier, Genève, Switzerland). Superoxide dismutase activity was assessed by in-gel zymography in the following way: the protein content present in approximately 15–20 mL of each cell-free growth medium (of cultures grown to a chlorophyll *a* concentration of 7–10 µg mL^−1^) and 100–550 µg of total protein were separated by electrophoresis on 10% (w/v) native-acrylamide gels. After electrophoresis, the gel was incubated 20 min in a 2.5 mM nitroblue-tetrazolium solution, followed by a 15-min incubation in SOD developing solution (36 mM potassium phosphate buffer, pH 7.8; 28 mM tetramethylethylenediamine and 86 µM riboflavin; for inhibition of the Fe containing SOD, 5 mM H_2_O_2_ was added to the SOD developing solution according to [41]). The gel was then exposed to a 60-W light source until full development. For catalase activity assessment, global catalase activity was determined following the H_2_O_2_ dissociation by measuring absorbance at 240 nm. In brief, between 300 and 450 µg of total protein or the protein content present in approximately 10–20 mL of culture were mixed in a quartz cuvette with 50 mM potassium phosphate buffer (pH 7.0) and H_2_O_2_ to a final concentration of 20 mM. The reaction was then followed spectrophotometrically at 240 nm for 3 min. Catalase activity is expressed as units per mg of total protein (when cell-free extracts were used) or units per µg of chlorophyll *a* (in the case of exoproteome samples), defining a unit as the amount of enzyme that catalyses the dissociation of 1 µmol of H_2_O_2_ per minute at pH 7.0 at room temperature. For both SOD and catalase activity determinations, protein samples obtained from at least three biological replicates were used.

Determination of reactive oxygen species (ROS): To determine the total amount of ROS found in BG11, BG11_0_ and BG11_0_ + NH_4_^+^, the general oxidative stress indicator 2’,7’-dichlorodihydrofluorescein diacetate (H_2_DCF-DA) (Life Technologies, Carlsbad, CA, USA) was used. When used in intact cells, intracellular esterases cleave the ester groups and remove the acetate, readily converting the non-fluorescent molecule H_2_DCF-DA to carboxy-dichlorofluorescein (DCF), a green-fluorescent form of the molecule upon oxidation by the activity of ROS. To obtain DCF *in vitro*, H_2_DCF-DA was hydrolysed with 0.01 M NaOH for 30 min at 37 °C in the dark [42]. Autoclaved BG11, BG11_0_ and BG11_0_ + NH_4_ media were kept sterile in the same conditions as cultures of *Anabaena* sp. PCC 7120, *i.e.*, in glass gas washing bottles with aeration, at 28 °C, under a continuous light regime of 30–40 µmol photons m^−2^ s^−1^. Samples of each sterile medium were collected and loaded on 96-well microtiter plates and mixed with DCF to a final concentration of 5 µM. ROS levels of medium samples supplemented with 0.5 mM H_2_O_2_ were also determined by DCF fluorescence in the same microtiter plate. After the addition of DCF, plates were incubated 1 h at 30 °C in the dark, followed by determination of DCF fluorescence at 528/20 nm after excitation at 485/20 nm on a Synergy 2 multi-mode microplate reader (BioTek, Winooski, VT, USA).

Negative-staining transmission electron microscopy: For negative staining transmission electron microscopy, 10 µL of concentrated exoproteome samples were mounted on Formvar/carbon film-coated mesh nickel grids (Electron Microscopy Sciences, Hatfield, PA, USA) and left standing for 2 min. The liquid in excess was removed with filter paper, and 10 µL of 1% uranyl acetate were added on to the grids and left standing for 10 s, after which, liquid in excess was removed with filter paper. Visualization was carried out on a Jeol JEM-1400 at 80 kV.

## 3. Results and Discussion

### 3.1. The Exoproteome of *Anabaena* sp. PCC 7120

**Figure 1:**
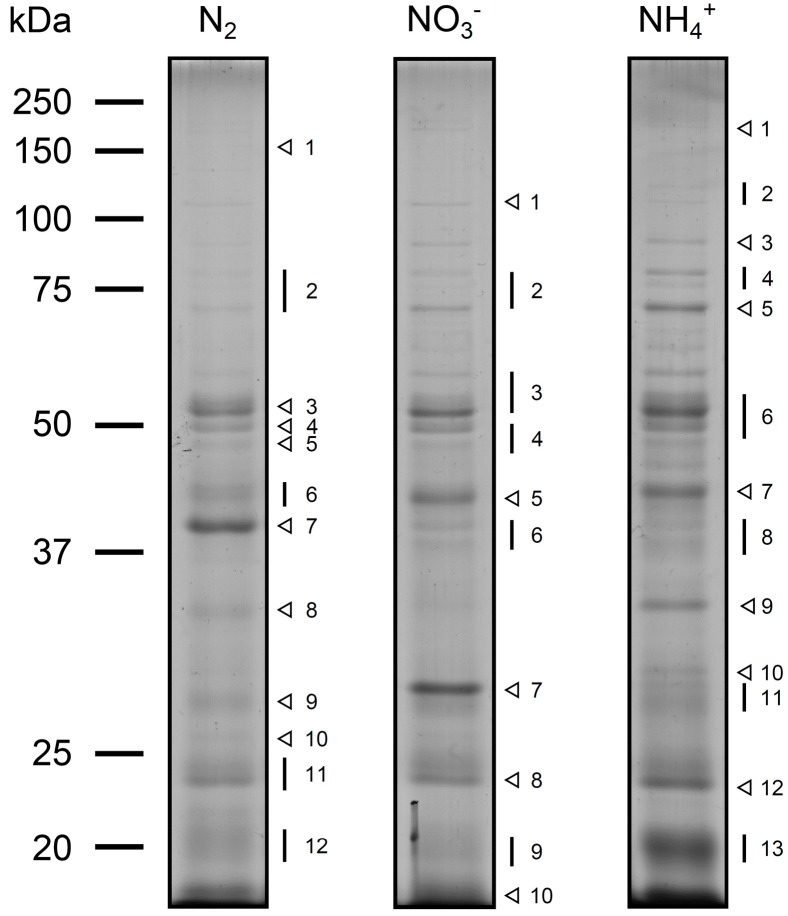
Exoproteome profiles of *Anabaena* sp. PCC 7120 cultivated under various growth conditions. Wild-type cells of *Anabaena* sp. PCC 7120 were grown in nitrogen-fixing conditions (N_2_) or in medium supplemented with nitrate (NO_3_^−^) or ammonia (NH_4_^+^). The protein content present in 5 mL of each cell-free growth medium was separated by SDS-polyacrylamide gel electrophoresis and the exoproteomes visualized by Coomassie Blue staining. Bands and gel areas selected for in-gel trypsin digestion and further protein identification by mass spectrometry are highlighted on the right of each panel by arrowheads and lines, respectively. Proteins identified in each band and the gel portion are listed in the Supplementary Information. The molecular masses of the Precision Plus Protein All Blue standard (Bio-Rad, Hercules, CA, USA) are indicated on the left.

Currently, in our group, we are identifying the exoproteome of various cyanobacterial strains, aiming at understanding the role of exoproteins on the cell structure and physiology. Briefly, the cells were grown under different conditions; samples were taken periodically during the culture growth and the growth medium was separated from the cells by mild centrifugation and filtration. The resulting cell-free medium was then concentrated by ultrafiltration (for details, see the Experimental Section). The exoproteome of *Anabaena* sp. PCC 7120 grown in medium with different combined nitrogen sources (nitrate or ammonia) or in nitrogen-fixing conditions separated by SDS-polyacrylamide gel electrophoresis is presented in Figure 1. The total amount of proteins accumulating in the growth medium, regardless of the cultivating conditions, is substantial, supporting the notion that *Anabaena* sp. PCC 7120 can indeed export and release proteins to the extracellular space with a specific function in that particular environment. This hypothesis gets further support from the observation that the exoproteome profiles in the three conditions tested present differential compositions, indicating that some proteins are specifically expressed in a given condition and could be exported to the extracellular milieu to fulfil a particular task.

When studying the exoproteome of a microorganism, the possible contribution of cell lysis or leakage to the whole exoproteome is an issue that deserves careful consideration. In the present work, this question was addressed experimentally, trying to minimize its contribution. For that purpose, growth medium from cultures of *Anabaena* sp. PCC 7120 cultivated in the three different growth conditions described was periodically sampled and the respective exoproteomes analysed (data not shown). This was done in order to evaluate whether some exoproteins could accumulate differentially in the extracellular space with respect to particular growth phases, but also to assess the contribution of cell lysis during the cultivation period (we hypothesized that as cultures get older, more cytoplasmic content ends up accumulating in the medium as a result of cell death and/or cumulative cell lysis). Hence, cultures that had reached a chlorophyll *a* concentration of approximately 8–10 µg mL^−1^ were then used for exoproteome analyses. Moreover, cells of *Anabaena* sp. PCC 7120 were also cultivated in different types of systems. Initially, cultivation was carried out in glass gas washing bottles with continuous aeration (see Experimental Section). However, since culture aeration may result in filament shearing and ultimately lead to mechanical cell lysis, we also grew *Anabaena* sp. PCC 7120 cultures in milder conditions, namely in 1-L Erlenmeyer flasks (with 300 mL of culture volume) in an orbital shaker with gentle shaking (100 rpm). However, no significant differences could be observed in the overall exoproteome composition between the various conditions as assessed by observation of colloidal Coomassie Brilliant Blue G-stained SDS-polyacrylamide gels (data not shown). Thus, cultivation in glass gas washing bottles was preferred due to the higher growth rates observed for *Anabaena* sp. PCC 7120.

In order to identify the proteins in the *Anabaena* sp. PCC 7120 exoproteomes, we started by performing mass spectrometric analyses directly on concentrated culture supernatants. However, given the high biomolecular heterogeneity of the cyanobacterial growth medium after cultivation, which is composed not only of proteins, but also of, e.g., extracellular polysaccharides [43,44], this approach resulted in poor protein identification. In fact, most of the results obtained could not be matched to any peptide. Thus, we decided to separate the various components of the concentrated supernatants by SDS-polyacrylamide gel electrophoresis and to proceed for protein identification by selecting Coomassie Blue-stained bands and/or gel portions that had been observed consistently across at least three biological replicates (see Figure 1).

Proteins identified in the extracellular milieu of *Anabaena* sp. PCC 7120 cells grown in nitrogen-fixing conditions or in medium supplemented with nitrate or ammonia are listed in Table 1 (for details, see the Supporting Information). Altogether, peptides from 139 different proteins, belonging to 16 functional categories, were identified.

**Table 1:** List of proteins identified in the exoproteome of *Anabaena* sp. PCC 7120 grown in nitrogen-fixing conditions or in media supplemented with nitrate or ammonia.

*Cyanobase* ^a^	*Annotation* ^b^	*Growth Condition* ^c^	*Functional Category* ^d^	*Previously Identified in* ^e^
All0004	ATP synthase gamma chain	NH_4_^+^	Photosynthesis and respiration	-
All0005	ATP synthase subunit alpha	N_2_, NH_4_^+^	Photosynthesis and respiration	*Anabaena*
All0167	Maltooligosyltrehalose synthase	NH_4_^+^	Other categories	-
All0168	Alpha-amylase	NO_3_^−^	Other categories	-
All0207	All0207 protein	NO_3_^−^	Conserved hypothetical protein	-
All0259	Cytochrome c-550	NH_4_^+^	Photosynthesis and respiration	-
All0268	All0268 protein	NH_4_^+^	Conserved hypothetical protein	-
All0275	Glycerophosphoryl diester phosphodiesterase	NO_3_^−^, NH_4_^+^	Other categories	*Anabaena*
All0458	Uncharacterized low temperature-induced protein all0458	NH_4_^+^	Conserved hypothetical protein	*Anabaena*
All0875	All0875 protein	N_2_, NH_4_^+^	Other categories	*Anabaena*
All1220	All1220 protein	NH_4_^+^	Conserved hypothetical protein	-
All1342	All1342 protein	NO_3_^−^	No similarity	-
All1380	All1380 protein	NO_3_^−^, NH_4_^+^	No similarity	-
All1683	Phosphoserine aminotransferase	N_2_	Amino acid biosynthesis	-
All1750	All1750 protein	NO_3_^−^	No similarity	-
All1951	Substrate-binding protein of ABC transporter	N_2_, NO_3_^−^, NH_4_^+^	Transport and binding proteins	-
All2105	FMN-dependent NADH-azoreductase	NH_4_^+^	Fatty acid, phospholipid and sterol metabolism	*Anabaena*
All2108	All2108 protein	NO_3_^−^	Conserved hypothetical protein	-
All2315	Ketol-acid reductoisomerase	NO_3_^−^, NH_4_^+^	Amino acid biosynthesis	-
All2316	Aldo/keto reductase	NH_4_^+^	Other categories	-
All2375	All2375 protein	NH_4_^+^	Transport and binding proteins	-
All2425	All2425 protein	NH_4_^+^	No similarity	-
All2453	Cytochrome b6-f complex iron-sulphur subunit 1	NH_4_^+^	Photosynthesis and respiration	-
All2498	N-acetyl-gamma-glutamyl-phosphate reductase 2	NH_4_^+^	Transport and binding proteins	-
All2533	Prolyl endopeptidase	N_2_, NH_4_^+^	Translation	-
All2563	Transaldolase	N_2_, NO_3_^−^, NH_4_^+^	Energy metabolism	*Anabaena*
All2567	Probable phosphoketolase 2	NH_4_^+^	Conserved hypothetical protein	-
All2655	All2655 protein	NH_4_^+^	No similarity	-
All2843	Alkaline phosphatase	NO_3_^−^, NH_4_^+^	Other categories	*N. commune*
All3149	All3149 protein	NO_3_^−^, NH_4_^+^	Conserved hypothetical protein	-
All3325	All3325 protein	NH_4_^+^	Conserved hypothetical protein	-
All3538	Enolase	N_2_, NH_4_^+^	Energy metabolism	*N. punctiforme*, *Anabaena*
All3556	Succinate-semialdehyde dehydrogenase	N_2_, NO_3_^−^	Energy metabolism	-
All3643	All3643 protein	NH_4_^+^	No similarity	-
All3653	Allophycocyanin subunit alpha-B	NH_4_^+^	Photosynthesis and respiration	-
All3791	Ribonuclease D	NH_4_^+^	Transcription	-
All3797	Beta-Ig-H3/fasciclin (Fragment)	N_2_, NH_4_^+^	Conserved hypothetical protein	*Anabaena*
All3909	Uroporphyrinogen decarboxylase	NO_3_^−^, NH_4_^+^	Biosynthesis of cofactors, prosthetic groups, and carriers	-
All3964	Phosphoglucomutase/phosphomannomutase	N_2_, NO_3_^−^, NH_4_^+^	Central intermediary metabolism	*Anabaena*
All3984	All3984 protein	N_2_	Conserved hypothetical protein	*N. punctiforme*
All4038	All4038 protein	NH_4_^+^	No similarity	-
All4050	All4050 protein	NH_4_^+^	Conserved hypothetical protein	*Anabaena*
All4121	Ferredoxin--NADP reductase	N_2_, NO_3_^−^, NH_4_^+^	Photosynthesis and respiration	*Anabaena*
All4131	Phosphoglycerate kinase	N_2_, NO_3_^−^, NH_4_^+^	Regulatory functions	*N. commune*, *N. punctiforme*, *Anabaena*
All4145	All4145 protein	NH_4_^+^	Other categories	-
All4191	DNA-directed RNA polymerase subunit alpha	NH_4_^+^	Transcription	-
All4214	50S ribosomal protein L4	NH_4_^+^	Translation	-
All4287	Peptidyl-prolyl cis-trans isomerase B	NH_4_^+^	Translation	*N. punctiforme*
All4388	All4388 protein	N_2_	Conserved hypothetical protein	-
All4464	Phosphoadenosine phosphosulfate reductase	NO_3_^−^, NH_4_^+^	Amino acid biosynthesis	-
All4499	All4499 protein	N_2_, NO_3_^−^	Conserved hypothetical protein	-
All4539	l-sorbosone dehydrogenase	N_2_, NO_3_^−^, NH_4_^+^	Other categories	*N. punctiforme*
All4563	Fructose-bisphosphate aldolase	NH_4_^+^	Other categories	-
All4575	Phosphate-binding periplasmic protein of phosphate ABC transporter	N_2_, NO_3_^−^, NH_4_^+^	Transport and binding proteins	-
All4749	All4749 protein	NO_3_^−^, NH_4_^+^	Conserved hypothetical protein	-
All4906	Phosphoglycerate mutase	N_2_	Energy metabolism	-
All4968	Glutathione reductase	N_2_, NO_3_^−^	Biosynthesis of cofactors, prosthetic groups, and carriers	*Anabaena*
All4985	Sucrose synthase	NH_4_^+^	Energy metabolism	-
All5039	ATP synthase subunit beta	N_2_, NO_3_^−^, NH_4_^+^	Photosynthesis and respiration	*Anabaena*
All5062	Glyceraldehyde-3-phosphate dehydrogenase 2	N_2_, NH_4_^+^	Photosynthesis and respiration	-
All7614	All7614 protein	N_2_, NO_3_^−^	Conserved hypothetical protein	-
All7633	All7633 protein	NO_3_^−^, NH_4_^+^	Conserved hypothetical protein	-
Alr0020	Phycobiliprotein ApcE	NH_4_^+^	Photosynthesis and respiration	-
Alr0021	Allophycocyanin subunit alpha 1	N_2_, NO_3_^−^, NH_4_^+^	Photosynthesis and respiration	*N. punctiforme*, *Anabaena*
Alr0022	Allophycocyanin subunit beta	N_2_, NO_3_^−^, NH_4_^+^	Photosynthesis and respiration	*Anabaena*
Alr0051	IMP dehydrogenase	NO_3_^−^	Purines, pyrimidines, nucleosides, and nucleotides	-
Alr0069	Ribonuclease PH	NH_4_^+^	Transcription	-
Alr0132	Alr0132 protein	NO_3_^−^, NH_4_^+^	Conserved hypothetical protein	*Anabaena*
Alr0140	Periplasmic oligopeptide-binding protein of oligopeptide ABC transporter	N_2_, NO_3_^−^, NH_4_^+^	Transport and binding proteins	-
Alr0169	Cyclomaltodextrin glucanotransferase	NH_4_^+^	Other categories	-
Alr0237	Probable cytosol aminopeptidase	N_2_, NH_4_^+^	Translation	-
Alr0267	Alr0267 protein	N_2_, NO_3_^−^	No similarity	*N. punctiforme*, *Anabaena*
Alr0474	Alr0474 protein	NO_3_^−^, NH_4_^+^	No similarity	-
Alr0523	Phycoerythrocyanin subunit beta	N_2_, NO_3_^−^, NH_4_^+^	Photosynthesis and respiration	*N. commune*
Alr0525	Phycobilisome 34.5 kDa linker polypeptide, phycoerythrocyanin-associated, rod	NO_3_^−^	Photosynthesis and respiration	*N. commune*
Alr0528	C-phycocyanin subunit beta	N_2_, NO_3_^−^, NH_4_^+^	Photosynthesis and respiration	*N. punctiforme*, *Anabaena*
Alr0529	C-phycocyanin alpha chain	N_2_, NO_3_^−^, NH_4_^+^	Photosynthesis and respiration	*N. punctiforme*, *Anabaena*
Alr0530	Phycobilisome 32.1 kDa linker polypeptide, phycocyanin-associated, rod	N_2_, NO_3_^−^, NH_4_^+^	Photosynthesis and respiration	*N. commune*
Alr0534	Phycobilisome rod-core linker polypeptide CpcG1	NO_3_^−^, NH_4_^+^	Photosynthesis and respiration	*N. commune*
Alr0608	Nitrate transport protein NrtA	N_2_, NO_3_^−^, NH_4_^+^	Amino acid biosynthesis	*Anabaena*
Alr0782	Ribulose-phosphate 3-epimerase	NH_4_^+^	Central intermediary metabolism	*N. commune*, *Anabaena*
Alr0834	Porin major outer membrane protein	N_2_, NO_3_^−^	Cell envelope	-
Alr0880	Oligopeptidase A	NH_4_^+^	Translation	-
Alr0996	Protease	N_2_	Translation	-
Alr1004	Alanine--glyoxylate aminotransferase	N_2_	Amino acid biosynthesis	-
Alr1050	Glucose-6-phosphate isomerase	N_2_, NO_3_^−^, NH_4_^+^	Energy metabolism	*Anabaena*
Alr1080	Acetylornithine aminotransferase	N_2_	Amino acid biosynthesis	-
Alr1299	Phosphoribosylglycinamide formyltransferase 2	N_2_	Purines, pyrimidines, nucleosides, and nucleotides	-
Alr1310	Alr1310 protein	NH_4_^+^	Conserved hypothetical protein	-
Alr1313	3-isopropylmalate dehydrogenase	NH_4_^+^	Amino acid biosynthesis	-
Alr1329	Alr1329 protein	N_2_	No similarity	-
Alr1348	Ferredoxin-sulphite reductase	NO_3_^−^	Other categories	-
Alr1362	Alr1362 protein	NO_3_^−^	Other categories	-
Alr1364	Alr1364 protein	NH_4_^+^	Conserved hypothetical protein	-
Alr1381	Calcium-dependent protease	NO_3_^−^	Translation	-
Alr1520	Alr1520 protein	NO_3_^−^	Conserved hypothetical protein	-
Alr1524	Ribulose bisphosphate carboxylase large chain	N_2_, NO_3_^−^	Photosynthesis and respiration	*N. punctiforme*
Alr1548	Alr1548 protein	NO_3_^−^, NH_4_^+^	Conserved hypothetical protein	-
Alr1742	Chaperone protein DnaK2	N_2_, NH_4_^+^	Cellular processes	-
Alr1834	Alr1834 protein	N_2_, NO_3_^−^, NH_4_^+^	Transport and binding proteins	-
Alr1965	ATP phosphoribosyltransferase	NO_3_^−^, NH_4_^+^	Other categories	-
Alr2190	Alpha-amylase	N_2_, NO_3_^−^	Other categories	-
Alr2313	Alr2313 protein	NO_3_^−^, NH_4_^+^	No similarity	-
Alr2328	Glutamine synthetase	N_2_, NO_3_^−^, NH_4_^+^	Amino acid biosynthesis	*N. punctiforme*, *Anabaena*
Alr2535	Branched-chain amino-acid ABC transport system periplasmic binding protein	N_2_	Transport and binding proteins	-
Alr2709	Alr2709 protein	N_2_	Conserved hypothetical protein	-
Alr2771	Dihydroxy-acid dehydratase	NO_3_^−^	Amino acid biosynthesis	*Anabaena*
Alr2877	Bicarbonate transport bicarbonate-binding protein	N_2_, NO_3_^−^, NH_4_^+^	Transport and binding proteins	-
Alr2887	Alr2887 protein	N_2_, NO_3_^−^	Conserved hypothetical protein	-
Alr2938	Superoxide dismutase	N_2_, NO_3_^−^, NH_4_^+^	Cellular processes	*N. commune*, *N. punctiforme*, *Anabaena*
Alr2948	Alr2948 protein	NO_3_^−^, NH_4_^+^	Other categories	-
Alr2973	Glucokinase	NH_4_^+^	Energy metabolism	-
Alr3090	Alr3090 protein	NH_4_^+^	Conserved hypothetical protein	*Anabaena*
Alr3344	Transketolase	N_2_, NH_4_^+^	Other categories	-
Alr3402	Nucleoside diphosphate kinase	NH_4_^+^	Purines, pyrimidines, nucleosides, and nucleotides	-
Alr3539	Alr3539 protein	N_2_, NO_3_^−^, NH_4_^+^	No similarity	*Anabaena*
Alr3588	Alr3588 protein	NO_3_^−^, NH_4_^+^	No similarity	*Anabaena*
Alr3607	Alr3607 protein	NO_3_^−^, NH_4_^+^	No similarity	-
Alr3608	Alr3608 protein	N_2_	Other categories	-
Alr3659	Alr3659 protein	N_2_, NH_4_^+^	Energy metabolism	*N. punctiforme*
Alr3808	Nutrient stress-induced DNA-binding protein	N_2_, NH_4_^+^	Other categories	*N. commune*, *Anabaena*
Alr4072	Alr4072 protein	N_2_, NO_3_^−^, NH_4_^+^	Other categories	-
Alr4123	Phosphoribulokinase	N_2_, NH_4_^+^	Photosynthesis and respiration	-
Alr4238	Alr4238 protein	NH_4_^+^	Other categories	*Anabaena*
Alr4385	Triosephosphate isomerase	NH_4_^+^	Energy metabolism	-
Alr4448	Endo-1,4-beta-xylanase	N_2_	Other categories	-
Alr4550	Uncharacterized protein alr4550	N_2_, NO_3_^−^, NH_4_^+^	Conserved hypothetical protein	*N. punctiforme*, *Anabaena*
Alr4641	Peroxiredoxin	NO_3_^−^, NH_4_^+^	Cellular processes	*Anabaena*
Alr4745	Dihydrolipoyl dehydrogenase	N_2_, NO_3_^−^, NH_4_^+^	Energy metabolism	-
Alr4794	Alr4794 protein	NO_3_^−^, NH_4_^+^	Conserved hypothetical protein	*Anabaena*
Alr4853	Aspartate aminotransferase	N_2_	Amino acid biosynthesis	-
Alr4907	Ornithine carbamoyltransferase	N_2_, NO_3_^−^, NH_4_^+^	Amino acid biosynthesis	-
Alr4976	Phosphodiesterase/alkaline phosphatase D	NO_3_^−^	Other categories	*N. commune*
Alr4979	Alr4979 protein	NH_4_^+^	Conserved hypothetical protein	*Anabaena*
Alr5103	ll-diaminopimelate aminotransferase 1	N_2_, NO_3_^−^	Other categories	*Anabaena*
Alr5182	Oxidoreductase	NH_4_^+^	Other categories	-
Alr7261	Alr7261 protein	N_2_, NO_3_^−^, NH_4_^+^	Other categories	-
Alr7346	Alr7346 protein	N_2_, NH_4_^+^	No similarity	-
Alr7524	Alr7524 protein	N_2_, NO_3_^−^, NH_4_^+^	Conserved hypothetical protein	-

^a^ Protein IDs according to Cyanobase (http://genome.microbedb.jp/cyanobase); ^b^ protein annotation according to the UniProt database (http://www.uniprot.org/); ^c^ indicates in which growth condition a particular protein has been identified, but should not be considered as a reference to whether that protein is found in that condition only; ^d^ functional category of each exoprotein, as found in Cyanobase (http://genome.microbedb.jp/cyanobase); ^e^ proteins (or orthologues) that have already been identified in previously-studied cyanobacterial exoproteomes or secretomes are highlighted; *N. commune*, *Nostoc commune* DRH1 [27]; *N. punctiforme*, *Nostoc punctiforme* PCC 73102 [30]; *Anabaena*, *Anabaena* sp. PCC 7120 [26].

In Section 3.1.1 and Section 3.1.2, a detailed analysis and discussion of the different proteins identified is presented, followed by some considerations regarding non-classical protein secretion, namely via outer membrane vesicle formation.

#### 3.1.1. Analysis of the *Anabaena* sp. PCC 7120 Identified Exoproteins

One question to bear in mind when analysing in detail the exoproteome composition of *Anabaena* sp. PCC 7120 is to know how a particular protein reached the extracellular milieu. Different scenarios may be considered: within the whole group of exoproteins, some may reach the extracellular space via active translocation across the cell wall and accumulate in this environment, because they play a role there. Others may also be translocated across the outer membrane, but instead are structural parts of the cell wall itself (or are involved in cell wall biogenesis) and are released to the extracellular milieu as a result of cell wall turnover. In addition, outer membrane and periplasmic proteins may also contribute to the exoproteome composition as a result of, e.g., passive protein leakage during cell division and cytokinesis, or by rupture of filament integrity, or even by active disposal of non-functional proteins/enzymes. Ultimately, cytoplasmic proteins may also contribute significantly to the composition of the exoproteome: cell death and mechanical shearing may lead to cell lysis. In that case, proteins that do not have a specific function in this environment may simply accumulate outside of the cells during cultivation, because they are relatively stable to proteolysis. However, in this work, knowing exactly how the identified exoproteins ended up in the extracellular milieu remains to be determined. Despite all of our efforts to experimentally minimize the contribution of proteins accumulating in the extracellular space as a result of cell lysis, we cannot exclude that some of the proteins identified are indeed cytoplasmic proteins. Nevertheless, the proteins listed in Table 1 are those identified from gel bands that could be detected consistently across several biological replicates. Thus, the analysis that will follow below regards them as exoproteins, even though the origin and extracellular function (if any) of most require additional experimental work.

**Figure 2:**
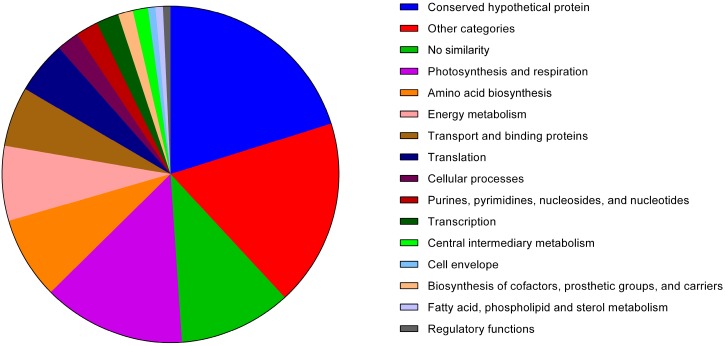
Absolute frequency of the various functional categories (as ascribed by Cyanobase, http://genome.microbedb.jp/cyanobase) of the proteins identified in the exoproteome of *Anabaena* sp. PCC 7120.

A distribution analysis of the proteins listed in Table 1 based on their respective functional categories (Figure 2) reveals that approximately 48% of the identified exoproteome is classified as “conserved hypothetical” (20%), presents no similarity to other proteins (10%) or falls into the “other categories” set (18%). The latter group of proteins comprises members associated with several unrelated processes, such as “transposon-related functions” and “adaptation and atypical conditions”, or are simply categorized as “other”. Thus, this group includes proteins with completely different functions and that share no obvious metabolic connection. Overall, the high frequency (48%) of identified proteins belonging to categories of unknown function further exposes our lack of knowledge regarding the impact of exoproteins and respective extracellular processes on the cell structure and physiology.

Among the identified extracellular proteins with assigned function, it is possible to find various examples of proteins involved in the degradation and processing of different types of biomolecules, including nucleic acids, along with interconversion and salvage of nucleosides (All3791, Alr0069, Alr7261, Alr3402, Alr0051, Alr1299, Alr1520), degradation of proteins and peptides, as well as processes regarding amino acid biosynthesis and processing (All2533, Alr0237, Alr0880, Alr0996, Alr1381, All4287, Alr1742, Alr4853, All2315, Alr1004, Alr1313, Alr2771, Alr0608, Alr1080, Alr2328 Alr4907, All1683, All4464), sugar breakdown and processing (Alr3608, Alr4448, All3964, All0167, All0168, All0875, Alr0169, Alr2190, All4539) and phosphor scavenge and transport (All0207, All2843, All4575, Alr4238, Alr4976). Detection of these proteins suggests that the processes carried out by *Anabaena* sp. PCC 7120 extracellularly are intimately associated with recycling of valuable nutrients and compounds present in the environment. These substrates may accumulate in the medium as the result of either active secretion across the cyanobacterial cell wall (e.g., extracellular polysaccharides) or by passive release as a consequence of cell lysis. In addition, proteins typically involved in ROS detoxification and redox homeostasis have also been identified, and a detailed analysis and discussion can be found in Section 3.2.

The three growth conditions used to cultivate *Anabaena* sp. PCC 7120 clearly induced the differential compositions of the exoproteomes (Figure 1). This observation indicates that some proteins are specifically expressed in a given condition and exported to the extracellular milieu to fulfil a particular task. However, the approach adopted and presented here does not allow performing direct comparisons of exoproteome differential compositions between culture conditions. In fact, relative abundance variances analyses should be preceded by better refined and resolved exoproteome separations, e.g., by two-dimensional gel electrophoresis, shotgun proteins and tagging [14,15,24] or label-free workflows [45]. Nevertheless, some differences can be easily identified: for example, Band 7 in the exoproteome of cells grown in BG11_0_ (Lane 1, N_2_), Bands 5 and 7 in the exoproteome of cells cultivated in BG11 (Lane 2, NO_3_^−^) and Bands 7, 9 and 13 in the exoproteome of cells grown in BG11_0_ + NH_4_^+^ (Lane 3, NH_4_^+^) contain proteins that accumulate in higher amounts relatively to the other growth conditions (Figure 1). Based on the number of unique peptides identified for each protein detected in each gel band/gel portion and on its respective protein sequence coverage (see the Supplementary Information), the most abundant protein in Band 7 in BG11_0_ is Alr0267. This exoprotein was recently shown to be involved in filament adhesion and aggregation in *Anabaena* sp. PCC 7120 cells grown in nitrogen-fixing conditions [34]. The protein was named HesF (for heterocyst specific attachment factor), and its secretion was shown to be dependent on the TolC-like HgdD protein, part of the type I secretion system [34]. Moreover, the respective gene transcription was shown to be highly upregulated upon a transition from non-nitrogen fixing to nitrogen fixing conditions [34], which could explain the protein’s higher abundance in BG11_0_ compared to BG11 or BG11_0_ + NH_4_^+^. The proteins identified in higher amounts in Band 5 in BG11 and with approximately the same abundance (according to the criteria outlined above; see the Supplementary Information) are Alr0608 (NrtA), the solute-binding component of the high-affinity nitrate ABC transporter [46], and Alr2877 (CmpA), the bicarbonate transport bicarbonate-binding protein [47]. In a medium containing nitrate, such as BG11, it is not surprising that NrtA is found in higher amounts than in any of the other two growth conditions. Interestingly, Band 7 in BG11 also contains high levels of NrtA, even though the molecular mass of this band is much lower than expected for the full-length NrtA (see the discussion below). Band 7 in BG11_0_ + NH_4_^+^, presents an intensity level comparable to Band 5 in BG11; however, the most abundant protein in the former is Alr2877. The gene encoding for protein Alr2877 has been shown to be regulated mainly by the inorganic carbon supply [47]. Nevertheless, it was also shown in the same work that expression of *alr2877* is significantly lower in *Anabaena* sp. PCC 7120 cells experiencing both inorganic carbon and combined nitrogen limiting conditions, as compared to cells growing in a medium limited in inorganic carbon, but replete with combined nitrogen [47]. Thus, these observations could account for the significant differences observed in the amounts of Alr2877 (CmpA) between the exoproteomes of cells cultivated in BG11 and BG11_0_ + NH_4_^+^ and the exoproteome of *Anabaena* sp. PCC 7120 cells grown in BG11_0_ (see Figure 1 and the Supplementary Information). Finally, and still in BG11_0_ + NH_4_^+^, Band 9 is majorly Alr0530 (phycobilisome linker polypeptide, phycocyanin associated) and Band 13 is composed mainly of Alr0022 (allophycocyanin subunit beta). The function of these two latter proteins on the extracellular space is unknown; however, it is interesting to notice that the concentrated supernatant of cells grown in BG11_0_ + NH_4_^+^ presented a bluish colour (data not shown), in agreement with the higher amounts of phycocyanin-related proteins. In general, when analysing the list of proteins identified in a given exoproteome, one question frequently raised concerns whether the proteins identified correspond to full-length, active enzymes or if they correspond to proteolytic fragments. To address that point, we have combined the analysis of the SDS-polyacrylamide gel electrophoresis separation of the exoproteome content (Figure 1) with the examination of the proteins identified in each band/area of the gel (see the Supplementary Information) and respective peptides detected by mass spectrometry (see the Supplementary Information). As mentioned above, Band 7 in BG11 is mostly represented by NrtA (Alr0608). NrtA is a lipoprotein that is tethered to the cytoplasmic membrane, facing the periplasm [46]. The NrtA predicted molecular mass is 48 kDa, while after cleavage of its signal peptide, it is estimated to be approximately 45 kDa [48], which matches well with the molecular mass of Band 5. However, Band 7 has an estimated molecular mass of 28 kDa. After analysing the NrtA, unique peptides detected in Band 5 the amino acid sequence between positions 87 and 422 were covered, while for Band 7, only peptides covering position 184 to 422 were obtained. Therefore, we believe that NrtA in Band 5 may correspond to the full-length protein, while in Band 7, NrtA is most likely an N-terminus truncated proteolytic fragment. Nevertheless, it remains elusive whether proteolysis occurred outside or inside the cell. Another interesting case is the unknown protein Alr0474, detected in the exoproteome of *Anabaena* sp. PCC 7120 cells grown in medium with nitrate (Bands 6 and 8). While Alr0474 (596 amino acids) peptides found in Band 6 cover amino acid positions 325 to 590, the ones identified in Band 8 correspond to positions 54 to 189. These results indicate that Alr0474 is likely to be released to the extracellular milieu and then be subjected to proteolysis. Future exoproteome studies will certainly help to clarify these matters and will further unveil the novel molecular mechanisms of protein turnover and recycling.

Something that cannot go unnoticed is the fact that some exoproteins are encoded by adjacent genes (located in the same or opposite strands) or genes that are located relatively close in the genome. These include cases in which the respective genes are located contiguously and in the same strand, such as All0004 and All0005, which are subunits of ATP synthase; All0167, All0168 and Alr0169 (the first two are encoded by genes located in one strand, while the latter is in the other) are all involved in carbohydrate metabolic processes; All2315 and All2316 are the ketol-acid reductoisomerase and the aldo/keto reductase, respectively; Alr0020 (phycobilisome subunit), Alr0021 and Alr0022 (both allophycocyanin subunits); and Alr0528, Alr0529 and Alr0530 are phycocyanin-related proteins. Alternatively, we have also registered cases in which the genes are contiguous, but divergently oriented in the genome: All1380 (no similarity) and Alr1381 (calcium-dependent protease); All2533 (prolyl endopeptidase) and Alr2535 (branched-chain amino-acid ABC transport system periplasmic binding protein), even though in this case, the short gene *asl2534* is located in between the respective genes; All3538 (enolase) and Alr3539 (unknown protein); All4121 (ferredoxin-NADP(+) reductase) and Alr4123 (phosphoribulokinase), even though the short gene *asl4122* is located in between the respective genes; All4906 (phosphoglycerate mutase) and Alr4907 (ornithine carbamoyltransferase); and Alr0267 and All0268, both unknown proteins. In addition, it is also possible to find proteins encoded by genes not located contiguously, but still in very close genomic proximity, such as All0875 and Alr0878, All2105 and All2108, All2563 and All2567, Alr1362 and Alr1364, Alr1520 and Alr1524 and Alr4976, Alr4979 and All4985. It is not uncommon to find contiguous or adjacent genes encoding extracellular proteins in other bacteria (e.g., [49]), but whether these observations in *Anabaena* sp. PCC 7120 represent fortuitous occurrences or reflect defined molecular mechanisms that may render gene transcription, protein translation and subsequent secretion more efficient remain to be determined.

Another interesting aspect worth mentioning regards the identification of proteins encoded by plasmid-borne genes. Proteins Alr7261 and Alr7346 (the respective genes are in the Alpha plasmid) and Alr7524, All7614 and All7633 (the respective genes are in the Beta plasmid) all belong to one of the categories “conserved hypothetical proteins”, “no similarity” or “other functions”. Even though the information available about these proteins is scarce, it is remarkable that based on the PSORTb subcellular localization prediction tool [39], Alr7261 is predicted to be an extracellular protein, while the others are predictably periplasmic (All7633), outer membrane (All7614), cytoplasmic (Alr7524) or unknown (Alr7346) proteins (see the Supplementary Information). In addition, Alr7261 and Alr7524 were found in the exoproteome of *Anabaena* sp. PCC 7120 cells grown under all tested conditions (Table 1). Despite the general lack of knowledge concerning proteins encoded by plasmid-borne genes, *alr7261* putatively encodes an extracellular sugar-non-specific nuclease NucA homologue (Cyanobase), whose most characterized member in *Anabaena* sp. PCC 7120 is All7362 [50,51]. Alternatively, All7614 has homology to carbohydrate-selective porins (OprB); members of this protein family have been shown in *Nostoc punctiforme* ATCC 29133 to function as sugar porins, being needed for the optimal uptake of both fructose and glucose [52].

The exoproteome of the cyanobacterium *N. punctiforme* PCC 73102 was recently reported [30]. In that work, the proteins accumulating in the extracellular milieu of cells grown with nitrate or in nitrogen-fixing conditions are presented, in a total of 33 proteins [30]. Furthermore, the secretome analysis of *Anabaena* sp. PCC 7120 was also recently presented by Hahn and co-workers [26], but they report the detection of secreted proteins (total of 50) from cells grown in a medium containing nitrate. In addition, the extracellular protein content (total of 13 proteins) of *Nostoc commune* DRH1, another filamentous, heterocyst-forming cyanobacterium, has also been studied, but here, the work was focused essentially on the protein content associated with its extracellular polysaccharide matrix [27]. Approximately 33% of the proteins identified in the present work have already been detected (or their orthologues) at least in one of the works mentioned above (see Table 1). The extended exoproteome profile presented here, as compared to the ones reported by others, may be accounted for in the three different growth conditions studied. Interestingly, two proteins have been identified in all of the studies focusing on the extracellular proteome of filamentous, nitrogen-fixing cyanobacteria: superoxide dismutase (Alr2938; see the discussion below) and phosphoglycerate kinase (All4131). The latter belongs to the “regulatory functions” category, even though it is a crucial enzyme in the energy metabolism. The presence of this enzyme in the extracellular space of three different cyanobacteria suggests a possible important function in that environment; however, it remains undetermined whether the enzyme is actually active and, if it is, what its function may be in such an environment.

Examining with further detail the work carried out on the secretome of *Anabaena* sp. PCC 7120, the closest to this work that we could find, a total of 72% of the proteins identified in that study could also be detected in this work. From those, it is worth referencing that proteins belonging to the categories “photosynthesis and respiration” (including ATP synthase and various phycobilisome subunits), “amino acid biosynthesis”, “central intermediary metabolism” (including phosphoglucomutase/phosphomannomutase and ribulose-phosphate 3-epimerase) and “energy metabolism” (transaldolase, glucose-6-phosphate isomerase and enolase) are found in both studies, supporting the notion that these proteins are indeed part of the exoproteome, despite being part of the primary metabolism of the organism. From the secreted proteins reported [26] that were not identified here, we can highlight All0070, a Mn-containing superoxide dismutase whose activity we were able to detect instead in the exoproteome (see below), a ferritin-like Dps protein (All1173), while here, we found instead the ferritin-like Dps proteins, All0458, Alr3808 and All4145; and the putative alkaline phosphatases, Alr2234 and Alr5291; while in our work, we identified All0207 and Alr4976.

#### 3.1.2. Membrane Vesicle Formation

When analysing the proteins identified in the various exoproteome samples of *Anabaena* sp. PCC 7120 (Table 1) and also reported by others [26,27,30], we could find several proteins predicted to be localized in the cytoplasm (Supplementary Information). Furthermore, few outer membrane proteins could also be observed in the exoproteome (Supplementary Information). At first glance, detection of such proteins in the extracellular space seems to be an indication of cell lysis and the passive release of proteins to the extracellular space, as already highlighted above. Nevertheless, identification of predicted cytosolic proteins in the extracellular milieu has been reported several times in a wide spectrum of bacteria, e.g., [53,54,55,56,57,58]. In the case of *Anabaena* sp. PCC 7120, the extracellular processes are so poorly studied and understood that it is possible that some of these proteins are indeed actively secreted [26], fulfilling a specific function yet to be described. Alternatively, it may also represent a mechanism for the removal of such proteins (or respective proteolytic degradation products) from the cytoplasm [26] or other subcellular locations (e.g., see NrtA, discussed above).

**Figure 3:**
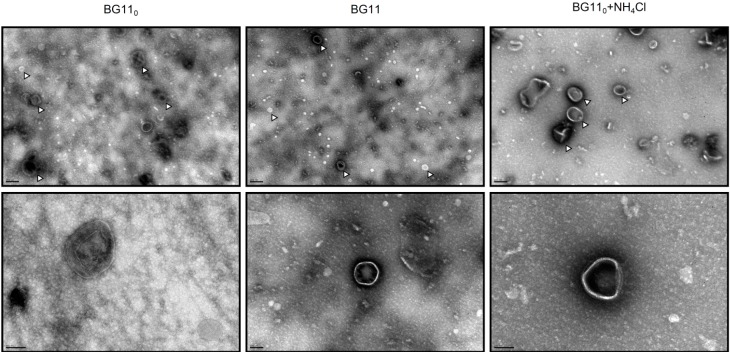
Negative staining electron micrographs of *Anabaena* sp. PCC 7120 concentrated exoproteome samples collected from cultures grown in nitrogen-fixing conditions (BG11_0_), or in medium supplemented with nitrate (BG11) or ammonia (BG11_0_ + NH_4_Cl). (**Top**) The presence and abundance of outer membrane vesicles (some highlighted by white arrowheads) in each sample (size bar, 200 nm); (**bottom**) the details of selected vesicles (size bar, 100 nm).

A good example to demonstrate how much is unknown in terms of extracellular processes is outer membrane vesicles (OMV) formation. A large number of heterotrophic Gram-negative bacteria naturally produce OMV, spherical bilayered vesicles released from the outer membrane, ranging in size from 50 to 250 nm in diameter [59]. Their suggested functions include toxin trafficking, DNA transfer and uptake, protein delivery and communication (reviewed by [59,60]). OMV formation by photoautotrophs was not described until earlier in 2014, when Biller and co-workers [61] were able to show it in cyanobacteria of the genera *Prochlorococcus* and *Synechococcus*. In that work, not only cultures of *Prochlorococcus* were shown to continuously release lipid vesicles containing proteins, DNA and RNA, but also that these vesicles could be found abundantly in coastal and open-ocean sea water samples. Most interestingly, *Prochlorococcus* vesicles were demonstrated to support the growth of heterotrophic bacteria, as well as to have the capacity of being recognized and infected by cyanophages [61]. Thus, as recognized by the authors, the ability to form vesicles by marine photoautotrophs adds another layer of complexity to the flow of information, energy and biomolecules in marine microbial ecosystems [61]. However, this may not be restricted to marine ecosystems, if OMV formation proves to be a common mechanism widespread to other cyanobacteria occupying different ecological niches. Proteomic analyses of *Prochlorococcus* MED4 vesicles identified a diverse set of proteins, including membrane nutrient transporters and porins, but also, predictably, soluble proteases and hydrolases and several proteins of unknown function [61]. Interestingly, from the 40 *Prochlorococcus* MED4 reported vesicle proteins, eight homologues could be found in the exoproteome of *Anabaena* sp. PCC 7120, including the porin TolC-like HgdD (Alr2887), the membrane transporters All4575 (phosphate), All1951 (ABC-type) and All4388 (putative polysaccharide exporter), the phosphoribosylglycinamide formyltransferase 2 (Alr1299), the ATP synthase α-subunit (All0005), the RuBisCO large subunit RbcL (Alr1524) and the hypothetical protein, All0268. Furthermore, it is also noteworthy that even though it was not possible to find the respective homologues for some of the *Prochlorococcus* MED4 vesicles’ proteins, related counterparts could be found for proteases (Alr1381, Alr0996), aminotransferases (All1683, Alr1004, Alr1080, Alr4853, Alr5103) and even ribosomal proteins (All4214) (see the Supplementary Information). Until now, there have been no reports describing the ability of *Anabaena* sp. PCC 7120 to form and release vesicles. Therefore, the different *Anabaena* sp. PCC 7120 concentrated exoproteome samples were subjected to negative staining transmission electron microscopy, in an attempt to find OMVs. Remarkably, the three samples presented numerous small spherical structures (Figure 3) that strongly resemble the membrane vesicles reported for *Prochlorococcus* [61].

This observation, together with the detection of membrane vesicles in the *Synechocystis* sp. PCC 6803 concentrated exoproteome (data not shown; [62]), suggests that outer membrane vesicle formation may represent an ability widely distributed in cyanobacteria. Hence, we propose that the set of membrane vesicles’ proteins identified in *Prochlorococcus* MED4 that could also be found in the exoproteome of *Anabaena* sp. PCC 7120 may be considered as part of the cyanobacterial membrane vesicle proteome core. However, *Prochlorococcus* and *Anabaena* are cyanobacterial genera that occupy very different ecological niches (marine *vs.* freshwater), present obvious morphological differences (unicellular *vs.* filamentous) and have different capabilities concerning cellular differentiation (no differentiated cells *vs.* heterocysts). In addition, the genome size of *Prochlorococcus* MED4 is approximately 2.4 Mbp, while the one of *Anabaena* sp. PCC 7120 is about three-times larger (7.2 Mbp). These differences may contribute to membrane vesicle proteomic variances and even account for an extended membrane vesicle proteome repertoire in organisms with larger genomes and more complex lifestyles. Therefore, it is possible that other proteins identified in the present work may also be originating from membrane vesicles. However, this will only be elucidated after the *Anabaena* sp. PCC 7120 outer membrane vesicles’ proteome becomes fully characterized. Therefore, in some prokaryotes, membrane vesicle formation represents a useful, highly efficient and effective, but yet largely overlooked, instrument of protein secretion: the fact that soluble proteins included in the OMV lumen, as well as bound to the OMV surface are co-released with insoluble material (lipids) may represent a protective mechanism, by which OMV-mediated transport can allow less stable molecules, such as protease-susceptible proteins, to reach their destination or behave like time-release capsules, which provide a beneficial activity at a later time [60]. Even more interesting is the capacity of OMV to be specifically targeted to a particular distal site through the binding specificity between surface-exposed bacterial proteins and environmental ligands or receptors [60].

### 3.2. Cyanobacterial Exoproteins Involved in Redox Homeostasis

One aspect on which we focused our attention was the identification in the extracellular milieu of proteins typically involved in ROS detoxification and redox homeostasis. The identified exoproteins include the Fe superoxide dismutase (Fe-SOD–Alr2938), a hypothetical Mn-catalase (Alr3090), a peroxiredoxin (Alr4641), the ferritin-like, nutrient stress-induced DNA binding proteins All0458, Alr3808 and All4145 and glutathione reductase (All4968).

**Figure 4:**
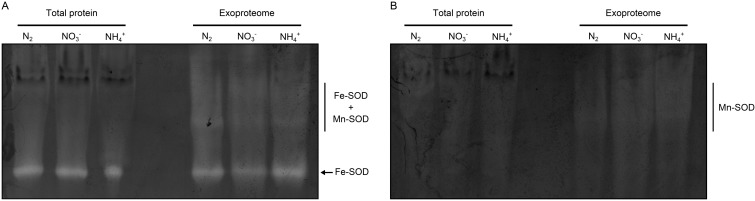
In-gel analysis of the SOD activities of *Anabaena* sp. PCC 7120. Cells were grown in nitrogen-fixing conditions (N_2_) or in medium with nitrate (NO_3_^−^) or ammonia (NH_4_^+^). Cell-free extracts were obtained from the collected cells, while the exoproteome was concentrated from the respective cell-free medium. Three hundred micrograms of total protein (total protein) and the protein content present in approximately 20 mL of culture (exoproteome) were separated by native-polyacrylamide gel electrophoresis. (**A**) Zymogram depicting total SOD activity. The Fe-SOD and Fe-SOD/Mn-SOD complexes’ activity bands are highlighted; (**B**) Zymogram showing the SOD activity bands, as a result of the specific inhibition of Fe-SOD with 5 mM H_2_O_2_ [41]. The Mn-SOD activity bands/smear are highlighted.

The ability of prokaryotes to export ROS protein scavengers to the extracellular space is well documented in the literature, particularly for pathogenic bacteria, such as mycobacteria [63,64], streptococci [65], *Campylobacter* [66] and *Corynebacterium* [58]. Since phagocytic cells produce reactive oxygen intermediates to kill invading bacteria, it is not surprising that these enzymes are important for virulence [63,64]. In contrast, reports describing the presence and activity of ROS detoxifying proteins in the extracellular space of cyanobacteria are limited to the work of Shirkey *et al.* [67]. In this work, the exudates (supernatant fractions) of desiccated colonies of *N. commune* ENG/1996 and cultures of *N. commune* DRH1 were shown to have high amounts of SOD, as well as high levels of enzyme activity [67]. It was also demonstrated that the extensive extracellular polysaccharide matrix of *N. commune* DRH1 generated superoxide radicals upon exposure to ultra-violet irradiation [67]. Hence, it was proposed that the SOD released by *N. commune* is crucial for the cyanobacterium to deal with the oxidative stress imposed by multiple cycles of desiccation and rehydration of the extracellular matrix during ultra-violet irradiation *in situ* [67]. *Anabaena* sp. PCC 7120 does not form a complex extracellular polysaccharide matrix similar to that described for *N. commune* strains, and still, SOD could be identified accumulating in the extracellular space. Fe-SOD was detected in the exoproteomes of cells grown under all tested conditions (Table 1) and not just in the exoproteome of cells grown under conditions eliciting the synthesis and secretion of large amounts of polysaccharides, as in nitrogen-fixing conditions. Therefore, we have decided to evaluate whether SOD activity could be detected in the three isolated exoproteomes. Our in-gel activity results clearly show the presence of SOD activity in all three isolated exoproteomes (Figure 4); in addition to the clear SOD activity band, other fainter bands/smear could also be observed (Figure 4A). We hypothesized that the additional fainter bands could be resulting from the activity of Fe-SOD (Alr2938) and Mn-containing SOD (All0070; not identified in this work) complexes. To test that hypothesis, the Fe-SOD activity was inhibited with the presence of H_2_O_2_; in such conditions, the intensity of the clearest SOD activity band completely disappeared, as well as part of the signal from the other fainter bands/smear, remaining however as part of the signal (Figure 4B). This result supports our initial suggestion that Fe-SOD and the Mn-containing SOD may indeed form complexes in the extracellular milieu.

In addition to the detection of Fe-SOD protein in the extracellular space of *Anabaena* sp. PCC 7120 (both in this work and in [26]) and in *N. commune* ENG/1996 and *N. commune* DRH1 [27,67], an SOD homologue was also identified recently in the exoproteome of *N. punctiforme* PCC 73102 [30]. Based on these results, it is tempting to assume that secretion of SOD may be a generalized mechanism adopted by cyanobacteria. The exoproteome of the unicellular, freshwater, non-nitrogen-fixing cyanobacterium, *Synechocystis* sp. PCC 6803, was isolated as described for *Anabaena* sp. PCC 7120, and the SOD activity was equally assessed by zymography. However, no SOD activity bands could be observed in *Synechocystis* sp. PCC 6803 (data not shown), suggesting that only a limited number of cyanobacterial species (thus far limited to filamentous and heterocyst-forming strains) are capable of exporting and accumulating active SOD in the extracellular milieu, at least under the conditions tested.

**Figure 5:**
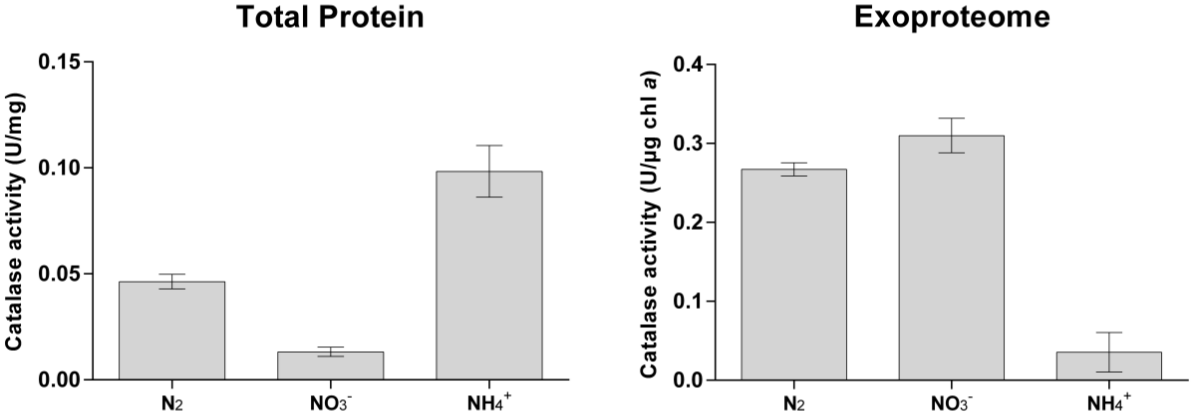
Catalase activity measured in cell-free extracts (total protein) or in concentrated exoproteomes of *Anabaena* sp. PCC 7120. Activities are expressed as units (defined as the amount of enzyme that catalyses the dissociation of 1 µmol of H_2_O_2_ per minute) per mg of total protein (**left**) or units per µg chlorophyll *a* (**right**).

We have also extended our analyses to evaluate catalase activity (Figure 5). Our results indicate that some factor in the *Anabaena* sp. PCC 7120 growth medium does possess the capacity of decomposing H_2_O_2_ (Figure 5), likely catalase. These results validate the identification of Alr3090 in the exoproteome of *Anabaena* sp. PCC 7120 and further support the suggestion that a complex oxidative stress defence mechanism exists extracellularly in this cyanobacterium. Nevertheless, total catalase activity levels determined here are low as compared to what is described for other cyanobacteria [68,69], even when using total protein extracts (Figure 5). However, catalase has been shown not to be the main mechanism to cope with H_2_O_2_ by the filamentous, heterocyst-forming cyanobacterium, *Anabaena* sp. PCC 7120; instead, peroxiredoxins are reported to be the main H_2_O_2_ detoxifying pathway [70], one of which (Alr4641) has been identified in the exoproteome of *Anabaena* sp. PCC 7120 (see the discussion below).

**Figure 6:**
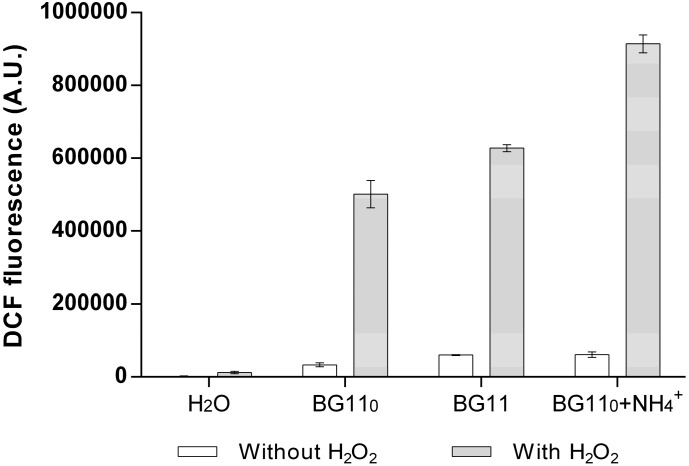
Reactive oxygen species (ROS) level determination in sterile cyanobacterial growth medium. Fluorescence of the molecular probe, DCF, which is directly related to its oxidation state and to the amount of ROS in the medium, was used to measure total ROS levels (A.U., arbitrary units). Cyanobacterial media BG11_0_, BG11 and BG11_0_ + NH_4_^+^ were kept sterile under the same conditions of temperature and aeration as cyanobacterial cultures. Water was used as the control. White bars, water or medium without H_2_O_2_; grey bars, water or medium with 0.5 mM H_2_O_2_. Given the heterogeneity in DCF fluorescence between different experiments, the data shown are from a single experiment, but the observed pattern is typical (at least three separate observations). The bar values are means, and the error bars correspond to the standard deviation from three technical replicates.

These results combined indicate that *Anabaena* sp. PCC 7120 is dealing with oxidative stress even outside of the cells. This notion may not be surprising for cyanobacteria in defined ecological circumstances, such as in mats, biofilms, symbiotic associations or even when free-living in freshwater bodies or marine ecosystems, since competition with other microorganisms for nutrients and even other cyanobacteria for light may involve ROS release and attack. However, in controlled laboratory conditions, in which the organism is cultivated axenically, it becomes difficult to interpret the observation that *Anabaena* sp. PCC 7120 exports and accumulates such high amounts of functional ROS detoxifying agents. Consequently, we became interested in analysing the ROS usually formed in normal growth medium under ordinary cultivating conditions, but without the presence of cells. Thus, the three media used to grow *Anabaena* sp. PCC 7120 (BG11_0_, BG11, BG11_0_ + NH_4_^+^) were kept sterile in the same conditions as cyanobacterial cultures. ROS levels were determined using the fluorescent redox indicator, DCF (dichlorodihydrofluorescein) (Figure 6).

Interestingly, as compared to distilled and deionized water, all three media presented higher levels of total ROS as indicated by a higher DCF fluorescence (Figure 6). In addition, all media presented approximately the same DCF fluorescence levels (white bars in Figure 6), suggesting that all present about the same amount of ROS. In order to monitor the probe’s responsiveness to the presence of ROS, 0.5 mM H_2_O_2_ were added exogenously to each medium and to water. The addition of H_2_O_2_ induced a much stronger fluorescence signal in any of the tested media as compared to water. Despite its evident utility and simplicity of use, DCF presents some disadvantages, one of which lies in its differential fluorescence response to various ROS (Life Technologies). This could account for the differences observed between water supplemented with H_2_O_2_ and all tested media supplemented with H_2_O_2_: since the reference BG11 medium is composed of minerals and several metals in solution [33], it is possible that H_2_O_2_ reacts with, e.g., iron (Fe^2+^) present in the medium in the so-called Fenton reaction, resulting in the formation of hydroxyl radicals. The DCF redox indicator has been shown to respond approximately 40-times more efficiently to the presence of hydroxyl radicals than to H_2_O_2_ (Life Technologies). Overall, these results show that ROS are generated naturally in ordinary cyanobacterial growth medium (something also observed for cultivating media of other organisms; [71] and the references therein) and that the interaction of H_2_O_2_ with medium components generates additional ROS. Moreover, these results also show that some cyanobacteria may release enzymes into the extracellular milieu to specifically detoxify exogenous ROS.

Regarding the other proteins involved in redox homeostasis identified in the exoproteome, the peroxiredoxin Alr4641 (2-Cys Prx) has been suggested to be the main mechanism by which *Anabaena* sp. PCC 7120 is capable of coping with H_2_O_2_, which presents abundant and sensitive intracellular levels of 2-Cys Prx, but low catalase activity [70]. Therefore, it may be possible that *Anabaena* sp. PCC 7120 secretes both catalase and 2-Cys Prx in order to cope efficiently with H_2_O_2_ exposure.

Furthermore, proteins All0458, Alr3808 and All4145 are ferritin-like, nutrient stress-induced DNA binding proteins (Dps). One of the main components providing structural integrity to biofilms, in addition to exopolysaccharides and proteins, is extracellular DNA [72]. Interestingly, Dps proteins have been shown to have an important role in *Campylobacter jejuni in vitro* biofilm formation [73], and Dps proteins have been identified in the exoproteomes of *Staphylococcus aureus* [74] and *Bacillus anthracis* [75] and even in the OMV of *Brucella melitensis* [76]. Thus, it is possible that *Anabaena* sp. PCC 7120 releases DNA to the extracellular space to promote biofilm formation, and Dps proteins are simultaneously translocated to protect extracellular DNA from structural damage elicited by oxidative stress.

Finally, the glutathione reductase (All4968) is the enzyme that catalyses the reduction of glutathione disulfide to glutathione with NADPH as the reducing cofactor. Glutathione is an extremely important antioxidant agent, preventing the damage of core cellular components caused by ROS. Interestingly, glutathione reductase has also been found in the exoproteome of the marine bacterium, *Roseobacter* sp. MED193 [77]. However, the presence of the protein in the extracellular milieu is rather puzzling: if glutathione may indeed play a role in maintaining redox homeostasis in the extracellular environment as a protection barrier in the cell vicinity against ROS (and, in fact, a bacterial glutathione transporter has been reported to export reductant to the periplasm of *Escherichia coli* [78]), it becomes difficult to interpret how glutathione reductase may be active when glutathione disulfide and reducing power in the form of NADPH are required. Additional work is needed to shed light on whether glutathione reductase is actively secreted by *Anabaena* sp. PCC 7120 and, if so, what may be the physiological impact/advantage of All4968 presence in the extracellular milieu of this cyanobacterium.

In general, it remains to be elucidated how these identified proteins involved in ROS detoxification and redox homeostasis reach the extracellular space, since no leader peptides can be detected in their primary amino acid sequences. One possibility could be that these proteins represent non-classical secreted proteins, and so, their secretory mechanisms remain elusive; alternatively, their transport to the extracellular milieu may be mediated by OMV, as described for SOD in *B. melitensis* [76] and *Acinetobacter baumannii* [79].

### 3.3. Modulating Cyanobacterial Protein Secretion: Future Perspectives

The competence for secreting proteins to the extracellular milieu is not exclusive to cyanobacteria, and it has already been used in certain prokaryotes to produce and secrete selected protein targets. In the case of applications where purified recombinant proteins are used directly, secretion of these proteins extracellularly could significantly reduce the complexity of a production process by eliminating the need for cell lysis and reducing the burden of removing host proteins [80]. Furthermore, secretion of highly expressed proteins minimizes the formation of inclusion bodies, aids in folding, allows for disulfide bond formation, reduces the effects of intracellular protein degradation and lessens the detrimental effects of cytotoxic proteins (reviewed by [80]).

Given the extraordinary physiological, metabolic and genetic qualities presented by cyanobacteria, we believe that if properly engineered, protein secretion by these photoautotrophs represents an attractive approach to further explore biotechnological solutions. In fact, the ability of cyanobacteria to use sunlight and carbon dioxide as energy and carbon sources, respectively, together with faster growth rates (compared to plants) and the relative ease with which they can be genetically engineered (compared to algae), make cyanobacteria stand out from all other organisms so far used in biotechnological applications [44,81]. In particular, cyanobacterial protein secretion may be a valuable alternative for solving various challenges in bioremediation, biomass recovery and biofuel production.

In addition to the strategies already in course for using cyanobacteria as efficient agents of bioremediation (e.g., making use of their exopolysaccharides [82]), cyanobacterial protein secretion could contribute significantly towards that goal; for example, nitrogen-fixing cyanobacteria could be genetically modified to express and secrete specific heavy metal chelators, contributing to detoxifying heavy-metal contaminated soils, as well as enriching their combined nitrogen content.

In order to reach commercially attractive figures in cyanobacteria-based biotechnological applications, the cultivation of cyanobacteria needs to be done at a large scale, which brings the issue of biomass recovery in large culture volumes. One of the strategies to separate growth medium from the valuable biomass has been to trigger cyanobacterial cells to adhere, aggregate and flocculate, easing its recovery. Some studies have shown that cyanobacterial cells adhesion and aggregation are facilitated by extracellular proteins [28,31,34]; thus, activation of those exoproteins expression and secretion with a precisely defined timing may aid in biomass recovery and reduce total biomass production costs.

In the case that whole microbial cells expressing recombinant proteins are used to interact with a polymeric material, extracellular secretion of proteins is necessary due to the inability of microbial cells to uptake polymer substrates [80]. Therefore, deconstruction of, e.g., polymeric lignocellulosic biomass to fermentable sugars is an important area with increasing interest in biofuel production from renewable resources. In this case, “polysaccharase” or “cellulase” secreting cyanobacteria would contribute to degrading these complex polymeric substances to simpler sugars; since cyanobacteria are capable of fixing their own carbon, products of lignocellulosic deconstruction would not be taken up by cyanobacteria, being instead fully available for other, more suitable fermenting bacteria that could then convert the recently released sugars into valuable biofuels.

In free-living nitrogen-fixing cyanobacteria, once atmospheric nitrogen is fixed to ammonia and assimilated to glutamine, it is believed to be partly converted to arginine, which is then polymerized into cyanophycin [83]. Cyanophycin synthesis after nitrogen fixation has been suggested to serve an important function by removing from solution the products of nitrogen fixation, which could have a negative feedback effect on nitrogenase [83,84]. Analogously, generating nitrogen-fixing cyanobacteria capable of secreting large amounts of a target protein would cause the organism to be drained off of fixed nitrogen and probably relieve repression and feedback on nitrogen fixation. Consequently, from the continuous protein secretion, one could expect higher nitrogen fixation rates and/or extended periods of nitrogenase activity to sustain growth, which would be an advantage for nitrogenase-based technological processes, such as the production of molecular hydrogen, a by-product of nitrogenase activity [85].

**Figure 7:**
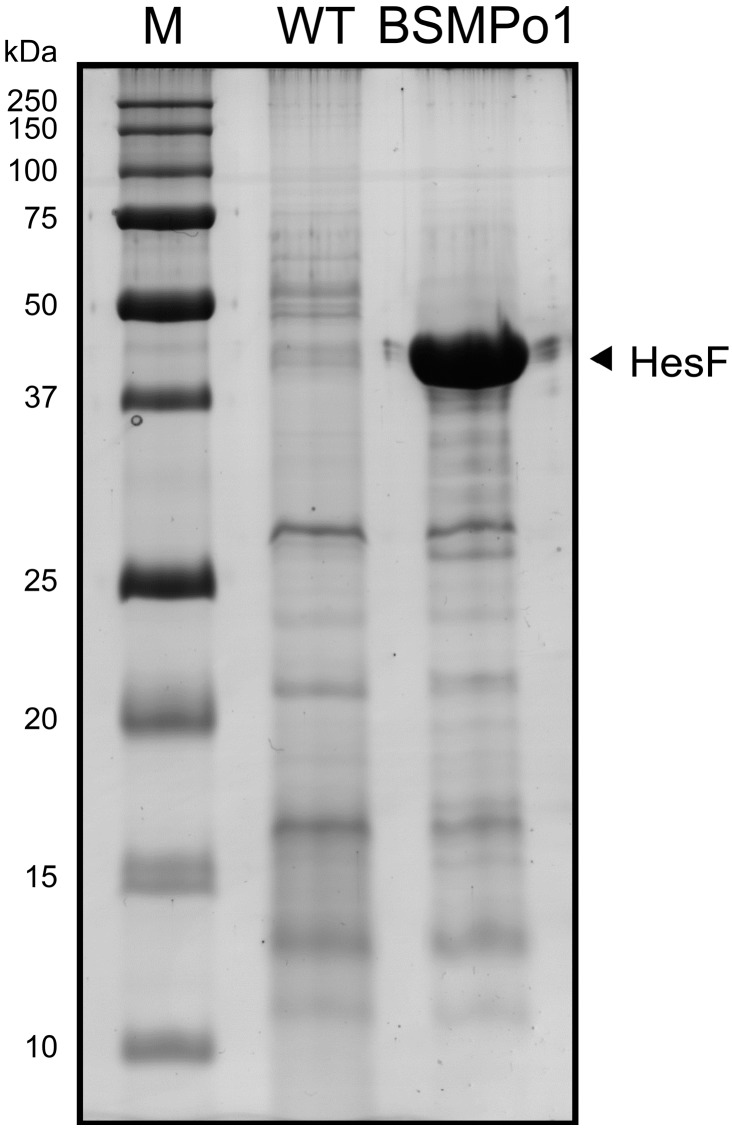
Exoproteome profiles of *Anabaena* sp. PCC 7120 wild-type (WT) and BSMPo1 [34] cultivated in BG11. The Coomassie-stained SDS-polyacrylamide gel shows the exoproteome of both strains grown for three days in a medium containing nitrate. The protein content present in approximately 3 mL of the growth medium was loaded on each lane. The molecular masses of the Precision Plus Protein All Blue standard (Bio-Rad), Lane M, are indicated on the left, while the arrowhead points to the overexpressed and secreted protein HesF.

Investigations recently carried out in our laboratory strongly indicate that there is room for engineering and modulating cyanobacterial protein secretion. As presented in Figure 7, the simple fusion of a nitrate inducible promoter with the exoprotein encoding gene, *hesF*, resulted in the overexpression of the protein, leading to an over-accumulation of HesF in the extracellular milieu. Furthermore, this result also highlights that even though a signal peptide could not be identified in the sequence of HesF, whatever addresses the protein for secretion seems to be highly efficient [34].

In order to explore protein secretion by cyanobacteria, a number of aspects need to be addressed and finely tuned. As for other bacterial systems, signal peptides or protein determinants that can address the translocation of a newly-synthesized protein from the cyanobacterial cytoplasm to the extracellular space represents one of the most crucial steps towards optimization of the secretion process. Signal peptides recognized by specific secretion systems are described for some bacteria (for a review, see [80]), and these could be tested and validated in cyanobacteria. Alternatively, in the specific case of HesF presented above, no signal peptide could be identified, and still, it must bear an efficient secretion determinant judging by the amounts of protein that accumulated in the growth medium. This represents a good example of how screening for novel signal peptides may open the door to more efficient secretion systems. Another aspect that deserves close attention when designing a protein secretion module is the selection of a promoter that will drive the transcription of the protein of interested coding gene. Cell-type specificity (in the case of cyanobacteria with the capacity of cellular differentiation), timing of expression and promoter strength are just a few of the points that need to be considered and coordinated with the particular protein to be secreted. Efforts to design suitable promoters for the most varied purposes and applications in cyanobacteria are being made [86,87] and will surely aid in engineering competent protein secretion microorganisms.

However, a few challenges loom ahead before successful implementation of engineered cyanobacterial cells to secrete selected target proteins can be attained. Vilhauer and co-workers showed that proteases are present and active in the extracellular space of *Nostoc punctiforme*, demonstrating as well that the extent of such proteolytic activity was dependent on the growth condition [30]. Thus, extracellular protease activity has to be considered and duly evaluated, so as to avoid poor protein recovery yields, low quality protein purifications or extracellular modules of low functionality, depending on the final application of the secreted protein. Still, this may be overcome by a balanced regulation between the rate of gene transcription, protein expression and protein secretion, as well as the rate of protein degradation, similarly to what happens in the cytoplasm of protein-(over)expressing bacteria. Moreover, the natural production and release of polysaccharides by cyanobacterial cells may also represent an obstacle towards the use of secreted proteins to fulfil particular goals.

Finally, the membrane vesicles that *Anabaena* sp. PCC 7120 is shown here to be able to produce represent an additional point of interest regarding protein secretion. The molecular mechanisms behind membrane vesicle formation, including the cellular structures and components responsible for packing those vesicles or even the determinants that govern what goes in and what remains out of the vesicles, are completely unknown in cyanobacteria. However, it is tempting to imagine the possibility of cyanobacteria forming membrane vesicles enriched in a target cargo that has been recombinantly produced: not only would this ease purification and increase the stability of the cargo, but also the membrane vesicles could act as delivery vectors. Certainly, many fundamental aspects need to be clarified before it becomes possible to be fully explored, but such a scenario is not completely unrealistic, since the delivery of foreign antigens by engineered outer membrane vesicle vaccines has already been demonstrated [88].

## 4. Conclusions

The exoproteome of the filamentous, heterocyst-forming cyanobacterium, *Anabaena* sp. PCC 7120, grown in nitrogen-fixing conditions or in medium supplemented either with nitrate or ammonia is presented in this work. Strikingly, as many as 139 proteins have been identified, belonging to 16 different functional categories. Proteins involved in oxidative stress detoxification and redox homeostasis are among the identified exoproteins, including SOD and catalase, whose activities we were able to detect in the concentrated supernatant. This suggests that *Anabaena* sp. PCC 7120 invests valuable cellular resources to eliminate reactive oxygen species, even outside of the cell. Furthermore, *Anabaena* sp. PCC 7120 is shown here, for the first time, to be able to release outer membrane vesicles that likely contribute to the whole exoproteome content. Finally, cyanobacteria seem to have the potential of becoming robust protein secretion factories, and future studies focusing on the cyanobacterial exoproteome will certainly contribute towards that goal.

## Supplementary Materials

Supplementary materials can be accessed at: http://www.mdpi.com/2075-1729/5/1/130/s1.
